# Identification of Serum Regression Signs in Infantile Hemangioma

**DOI:** 10.1371/journal.pone.0088545

**Published:** 2014-03-05

**Authors:** Daniela D'Arcangelo, Ezio M. Nicodemi, Stefania Rossi, Claudia Giampietri, Francesco Facchiano, Antonio Facchiano

**Affiliations:** 1 Istituto Dermopatico dell'Immacolata, IDI-IRCCS, Rome, Italy; 2 Divisione Chirurgia Plastica, Centro Anomalie Vascolari, Istituto Dermopatico dell'Immacolata, IDI-IRCCS, Rome, Italy; 3 Dipartimento Ematologia, Oncologia e Medicina Molecolare, Istituto Superiore di Sanità, Rome, Italy; 4 Department of Anatomy, Histology, Forensic Medicine and Orthopedics - Section of Histology and Medical Embryology, Sapienza University, Rome, Italy; University of Bari Medical School, Italy

## Abstract

Vessel proliferation underlies a number of serious pathological conditions. Infantile Hemangioma (IH) is a low-aggressive vascular tumor, interesting as an *in vivo* model of spontaneous tumor regression. Identifying mechanisms underlying IH spontaneous regression may then help to elucidate vessel-growth control, strongly deregulated in other serious conditions such as sarcoma, melanoma, diabetic retinopathy. The present study was aimed at identifying early regression indicators within hematological parameters. Thirty-four blood samples were collected from IH diagnosed babies (20-months median age), spontaneously regressing with age. Nineteen serum standard blood-tests were carried out using diagnostic reagents; in addition, serum-expression of 27 cytokine/chemokines was measured. Samples were divided in three age-groups, namely ≤12, 13 to 24 and >24 months-age, respectively. Red-cells count, Hemoglobin, Hematocrit, Neutrophils, Lymphocytes, MCP-1 and MIP-1beta were significantly different in the three age-groups, according to one-way ANOVA analysis. The same parameters showed a significant Pearson-correlation with age, supporting the direct link of age with IH-regression. ROC analysis showed that red-cells count, Hemoglobin, Hematocrit, MCP-1 and MIP-1beta levels significantly discriminate IH in the proliferating-phase from IH in the regressing-phase. Such data indicate for the first time that standard hematological tests and cytokine serum-expression values may effectively discriminate proliferating- from regressing-IH, unrevealing early regression signs, and demonstrate that standard blood-tests may have novel unsuspected diagnostic/prognostic relevance in altered vessel-growth conditions.

## Introduction

Neo-vascularisation is a key process regulating tumor growth, in fact the anti-angiogenic approach is a useful therapeutic strategy in many solid tumors. Therefore, investigating growth and regression of vascular tumors may help clarification of key mechanisms deregulated in cancer onset as well as in other serious diseases [1].

Investigating serum-soluble factors in children may represent a favourable condition, as compared to adults, due to the higher proportional mass of the disease as compared to the body weight. Infantile hemangioma (IH) is a vascular tumor with unique characteristics, particularly interesting from both clinical and biological point of views. Despite its low-aggressiveness it is of large interest since it may be considered as a human *in vivo* model of spontaneous cancer regression. Therefore a thorough investigation in such model may reveal mechanisms underlying its regression not evident in adults, where soluble factors are highly diluted in the blood.

IH represents the most frequent vascular tumor of early childhood [2], [3], occurring in 3 to 10% of white infants [4] mostly in Caucasian infants, female, premature, low-weight babies [5]. Such vascular lesions usually evolve with the baby age, from an initial proliferation phase toward a final regression phase [6]. Differently from other classical vascular malformations exhibiting developmental defects with a quiescent endothelium, IH grows by a clear hyperplastic mechanism [7]. In addition, classical vascular malformations are evident at the birth, while IH becomes manifest 1 week to 1 month after birth. A differential diagnosis of IH with vascular abnormalities or other serious vascular tumors is currently based on the inspection and clinical history; early molecular markers able to discriminate IH from other more serious diseases are currently lacking and may help the physician to plan the correct therapeutic approach.

A peculiar IH feature is the spontaneous regression over time. In hemangioma three phases occur within the 5^th^ to the 7^th^ year of age, namely proliferating phase (typically lasting up to the 9–12^th^ month of age), involuting phase and involuted phase. IH arises from multipotent stem cells leading to endothelial cells in the proliferating phase and to adipocytes during the involution/involuted phase and molecular mechanisms underlying hemangioma onset and involution are only partially defined [8]. Messenger RNA level of β-adrenoceptor has been indicated as possibly involved and such level may represent a potential molecular marker useful to discriminate IH from other vascular malformations [9]. Furthermore, Glucose transporter-1 (Glut-1) may also represent an useful diagnostic marker [10]. It is a transporter protein, commonly associated with erythrocytes, giving unique immunoreactivity in IH specimens and suggests a possible link of IH with placenta growth [11]. IH clinical features have been recently reviewed [12].

The IH growing phase lasts usually until about the 9^th^–12th month of life followed by a slow regression. In most cases by adolescence the tumor is completely regressed, appearing as a fibrotic mass with fatty components [13], therefore IH usually does not require specific surgical intervention. However, in about 20% of cases IH occurs in vital organs surrounding areas (e.g. airway, eyes or nasal region); in such cases a surgical or medical intervention is necessary. In other cases internal bleeding or ulceration must be avoided to rule out possible complications [14]. Propranolol, a non-selective inhibitor of β(1)- and β(2)-adrenoceptor has been recently introduced as medical intervention to treat severe proliferating IH; it may act mostly via a vasoconstrictor action on pericytes, however its exact mechanism of action is still poorly understood [15].

We have previously shown that blood itself may contain unsuspected prognostic or diagnostic information associated to serum parameters. For instance we showed that the fine balance of PDGF-BB and TGF-beta1 blood levels may represent an early predictive sign of kidney-transplant failure [16]. More recently we reported potential diagnostic relevance related to the presence of alpha-2-macroblbulin and apo-lipoproteins in the sera of melanoma patients [17]. We are currently further investigating the presence of specific markers with diagnostic relevance in melanoma patients sera. Finally, in collaboration with other groups, we demonstrated the association of a specific hematological cytokine-profile in chronic heart failure patients [18]. These, and many other, data fully support the hypothesis that specific hematological parameters, under particular conditions, may acquire unsuspected diagnostic/prognostic relevance. In the present study 34 blood samples were obtained from babies showing IH in the proliferating phase as well as in the involuting/involuted phase, and 46 different serum parameters were analyzed (including 19 standard hematological analyses such as total red blood cells, total white blood cells, Hemoglobin related values, leukocytes formula, plus other serum values such as 27 cytokine/chemokines serum expression levels).

The very low age made it difficult to recruit a larger number of patients. However, results presented in the current study show strong statistical significance, and represent the first study reporting an extensive serum profiling in IH, reporting indicators and molecules clearly related to IH regression.

## Materials and Methods

### Patient recruitment

This was a prospective study. Thirty four samples were analyzed in the present study. Twenty eight different babies with median age of 20 months, affected by IH, were consecutively recruited (from 2009–2012) at Centro Anomalie Vascolari of IDI-IRCCS, according to a protocol approved by the IDI-IRCCS Ethic Committee (protocol n. 2009-303/1), with written informed consent signed by the parent of the children enrolled. A total of 34 samples were collected from babies at different ages. Inclusion criteria were: IH diagnosis, no other evident pathologies. Sample size was taken as large as possible, given the objective difficulties at recruiting small babies, affected by a spontaneously regressing benign disease. Size of the hemangioma lesions ranged from 0.5 to 6.5 cm diameter. Each patient showed typically one major lesion and all patients showed a spontaneous regression without any systemic or topic treatment.

Data in the present manuscript are only reported in aggregated anonymous form.

### Blood collection and handling

All babies underwent blood collection; blood was carefully collected in a vial lacking any chemical or mechanical additive. Coagulation was allowed to naturally occur for two hours at room temperature. Blood was then centrifuged at 2500 rpm for 15 min; serum was collected and immediately frozen at −80°C in 100 µl aliquots. Aliquots were thawed, used once and never refrozen or re-used.

### Standard hematological analyses

Nineteen standard blood tests were carried out using diagnostic reagents at the diagnostic service of IDI-IRCCS hospital. Such analyses included total red blood cells, total white blood cells, total Hemoglobin content, Hematocrit (HCT), mean volume of red cells (MCV), mean cell Hemoglobin content (MCH), mean corpuscular Hemoglobin concentration (MCHC), Red blood cell distribution width (RDW), Hemoglobin Distribution Width (HDW), Platelets content, mean platelets volume (MPV), platelet hematocrit (PCT), platelet distribution width (PDW), Neutrophils %, Lymphocytes %, Monocytes %, Eosinophils %, Basophils %, Large Unstained Cells (LUC) %.

### Cytokine profiling

Serum expression of 27 human different cytokine/chemokines was measured with the 27-Bio-Plex assay kit (BioRad Laboratories, Milan, Italy), a magnetic-bead based multiplex immunoassay, carried out according to manufacturer guidelines. The following factors were measured:

IL-1ß, IL-1Ra, IL-2, IL-4, IL-5, IL-6, IL-7, IL-8, IL-9, IL-10, IL-12, IL-13, IL-15, IL-17, Eotaxin, FGF-2, G-CSF, GM-CSF, IFN-γ; IP-10; MCP-1, MIP-1α, MIP-1ß, PDGF-BB, RANTES, TNF-α, VEGF. Bio-Plex200 instrument, equipped with the Bio-Plex Manager Software 4.1, was used. Calibration, validation and fluidics washing were carried out before running the assays, as requested. Sample dilution (1∶4 in the sample-dilution buffer) and handling was carried out strictly according to manufacturer instructions. The logistic 5PL five-parameters not-linear regression type equation was carried out for raw data analysis, according to standard procedures in Bio-Plex Manager. Measurements were carried out in duplicates.

### Statistical analysis

Data were analyzed with GraphPad Prism for Windows, version 5.04 (GraphPad Software Inc.).

Samples were divided in three groups with similar numerosity: babies with IH in the proliferating phase (≤12 months; N = 10); babies with IH in the early regressing phase (age between 13 and 24 months; N = 12); babies with IH in the advanced regressing phase (age>24 months, N = 12). One-way ANOVA analysis was carried to measure the significant differences. Significant linear trends were also identified with a post hoc analysis by GraphPad Prism software.

In an additional statistical analysis the 46 parameters were directly analyzed as function of age according to Pearson correlation. Reference data-points for healthy babies were retrieved from the Mayo clinic site http://www.mayomedicallaboratories.com/test-info/pediatric/refvalues/reference.ph. Each data-point was exploded to three data-points containing the lowest, the median and the highest point, in order to obtain “distributed” data.

Finally, Receiver Operating Characteristic (ROC) analysis was also carried out to verify whether values were able to significantly discriminate proliferating IH samples (age≤12 months) from samples in the regressing phase (age>12 months). ROC analysis takes into account sensitivity and specificity accuracy. A significance threshold of p<0.05 was considered. Data were expressed as median ± Standard Error.

The present manuscript reports results obtained with parametric analyses since the normality test performed according to Kolmogorov-Smirnov (as well as to D'Agostino and Pearson test) computed a normal distribution for MIP-1ß, MCP-1, RBG, Hemoglobin, HCT, Neutrophils, Lymphocytes values. In order to take into account the relatively small sample size, the non parametric Kruskal-Wallis test was also carried out, as an alternative to the one-way ANOVA analysis. Results were similar to the parametric analyses.

## Results

### Samples characteristics

Table S1 reports the general characteristics of the population under study. Thirty four blood samples were collected from babies affected by IH with a median age of 20±4 months (11 male and 23 female, median age 18±7 months and 20±4, respectively). Samples were divided in groups according to the clinical phase: proliferating phase (≤12 months age), early regressing (involuting) phase (13 to 24 months age), and advanced regressing (involuted) phase (>24 months age). Table S1 also reports size and median age of each group. All recruited babies showed a spontaneous IH regression with age.

### Significant differences by one-way ANOVA, in serum profiling, as function of IH phase

Table 1 reports the 19 standard hematological values measured in the three groups. A one-way ANOVA analysis indicated strong and significant differences for 5 values, namely: red blood cells, HCT, Hemoglobin, Neutrophils % and Lymphocytes % (highlighted in grey). In all cases such values also show a significant increasing or decreasing linear trend (see right hand site of Table 1). Table 2 reports serum expression of 27 cytokine/chemokines (expressed in pg/ml) measured by a multiplex magnetic-beads based ELISA assay, in the same three age-groups. Significant differences, according to one-way ANOVA analysis, are highlighted in grey and indicate that PDGF-BB, IL-4, IL-17, MCP-1 and MIP-1ß show strongly significant differences within the groups. The most relevant and most significant changes were observed for MCP-1 and MIP-1ß showing also a significant linear decreasing trend (p = 0.0016 and p = 0.001, respectively), leading to about 50% reduction throughout the groups (see right hand site of Table 2). The significant linear trend indicated in Table 1 and Table 2 suggests a direct relation with the age and with the IH phase.

**Table 1:** Hematologic profile in hemangioma patients, in three age-groups.

	≤12 months N = 10	13–24 months N = 12	>24 months N = 12			
				Anova	Linear trend	
	AVG ± SE	AVG ± SE	AVG ± SE	*P*	*p*	
White cells/ml(*10^3^)	9.3±1.0	10.1±0.8	8.8±0.5	-	-	
Red cells/ml(*10^6^)	**4.3±0.1**	**5.0±0.1**	**5.1±0.1**	**0.002**	**0.0008**	↑
**Hemoglobin (g/dl)**	**11.5±0.2**	**12.5±0.3**	**13.1±0.2**	**0.0004**	**0.0001**	↑
**HCT (%)**	**32.3±0.7**	**36.9±1.0**	**38.3±0.6**	**0.0001**	**0.0001**	↑
MCV (f/L)	74.7±1.3	74.7±1.3	74.1±1.0	-	-	
MCH (pg)	25.9±0.7	25.6±0.6	25.1±0.3	-	-	
MCHC (g/dl)	35.2±0.4	33.8±0.4	33.9±0.4	-	-	
RDW (%)	12.8±0.2	12.7±0.3	12.4±0.2	-	-	
HDW (g/dl)	2.8±0.1	2.7±0.1	2.8±0.1	-	-	
Platelets/ml(*10^3^)	421.0±60	382.0±31	351.0±17	-	-	
MPV (fl)	8.5±0.4	7.3±0.3	7.5±0.3	-	-	
PCT (%)	0.4±0.1	0.3±0.0	0.3±0.0	-	-	
PDW (%)	38.6±1.7	35.9±1.3	37.1±1.0	-	-	
**Neutrophiles (%)**	**25.5±2.6**	**29.4±3.2**	**36.9±3.7**	**0.04**	**0.01**	↑
**Lymphocytes (%)**	**63.4±3.0**	**58.0±3.4**	**49.7±3.5**	**0.02**	**0.009**	↓
Monocytes (%)	6.0±0.7	5.6±0.4	6.6±0.5	-	-	
Eosinophils (%)	2.8±0.5	1.7±0.4	2.7±0.8	-	-	
Basofils (%)	0.4±0.1	0.6±0.1	0.6±0.1	-	-	
LUC (%)	3.3±0.2	3.7±0.2	3.2±0.4	-	-	

**Table 2:** Cytokines serum expression in IH patients*, in three age-groups.

	≤12 months N = 10	13–24 months N = 12	>24 months N = 12			
				Anova	Linear trend	
	AVG ± SE	AVG ± SE	AVG ± SE	*p*	*p*	
PDGF-BB	4940.5±301.0	4139.0±227.0	4823.5±192	0.03	-	
IL-1ß	46.5±4.3	46.5±4.1	48.5±2.9	-	-	
IP-10	534.5±230	469.0±275.0	665.5±157	-	-	
IL-1Ra	31.0±4.1	29.5±7.0	35.0±17.2	-	-	
IL-2	n.d.	n.d.	n.d.			
IL-4	119.5±7.0	103.0±5.9	120.8±4.9	0.03	-	
IL-5	34.0±51.0	32.0±2.7	33.3±2.2	-	-	
IL-6	43.0±6.1	37.0±8.6	38.5±6.8	-	-	
IL-7	47.8±4.8	49.5±4.6	41.8±3.4	-	-	
IL-8	96.8±16.5	72.0±2.9	92.5±18.9	-	-	
IL-9	n.d.	n.d.	n.d.			
IL-10	77.5±17.1	93.5±13.9	61.5±10.8	-	-	
IL-12(p70)	164.0±34.9	206.0±23.3	128.3±20.5	-	-	
IL-13	72.5±9.0	98.0±9.5	67.3±6.7	-	-	
IL-15	n.d.	n.d.	n.d.			
IL-17	129.5±11.5	88.5±10.3	117.5±9.2	0.03	-	
Eotaxin	22.5±16.2	n.d.	n.d.	-	-	
FGF-2	11.8±6.6	22.0±6.9	26.8±8.8	-	-	
G-CSF	39.5±2.4	31.0±4.9	33.8±1.4	-	-	
GM-CSF	n.d.	n.d.	n.d.			
IFN-γ	91.3±5.0	82.0±5.5	92.0±4.6	-	-	
**MCP-1**	**466.5±59.7**	**314.5±45.3**	**238.3±34.7**	**0.006**	**0.0016**	↓
MIP-1α	61.3±4.4	47.5±5.2	50.0±7.2	-	-	
**MIP-1ß**	**1404.0±145.0**	**801.5±101**	**779.0±63.6**	**0.0001**	**0.0001**	↓
RANTES	11610±205.0	12001±185	12030±114	-	-	
TNF-α	30.0±3.4	26.5±2.7	27.5±3.3	-	-	
VEGF	213±101	273±61.5	174.3±35.7	-	-	

* Values are expressed in pg/ml.

n.d. : not detected.

Expression of other key factors appears to be strongly changed in Table 2, although in a not-significant and not-linear way, for instance IP-10, IL-12, FGF-2 and VEGF. On the contrary, others appear almost identically expressed in the three groups (namely IL-5, IL-6, IL-7, G-CSF, IFN-γ, TNFα).

The caution in interpreting such data is mandatory, given the relatively small size of the groups, nevertheless the high statistical significance suggests that hematological profiles shown in Table 1 and Table 2 may represent signs related to the IH regression.

### Correlation with age of collection

The above reported analyses were carried out on samples divided in three age-groups with similar numerosity. We then wondered whether the hematological values under investigation show any linear (direct or inverse) relation with the age and a Pearson correlation analysis was performed.

Out of the 46 different parameters measured on IH samples, only 7 showed a significant correlation index. As reported in Table 3, these are the same 7 parameters showing a linear trend in Table 1 and Table 2. It should be underlined that none of these showed a significant correlation in the healthy reference values. “Normal” values for small babies are hard to collect, given also ethical concerns. We then obtained reference values from the literature (data form Mayo Clinic available at http://www.mayomedicallaboratories.com/test-info/pediatric/refvalues/reference.php).

**Table 3:** Correlation with age*.

	Pediatric healthy population §		Pediatric IH population ¶	
	R *	*p*	R	*p*
Red cells	0.2	n.s.	0.36	0.03
Hemoglobin	−0.3	n.s.	0.54	0.003
HCT	−0.2	n.s.	0.54	0.009
Neutrophils	0.09	n.s.	0.46	0.004
Lymphocytes	−0.1	n.s.	−0.47	0.004
MCP-1	n.a.		−0.35	0.05
MIP-1ß	n.a.		−0.45	0.008

* Correlation index R according to Pearson analysis and *p* value.

§ Values from Mayo Medical Laboratories at http://www.mayomedicallaboratories.com/test-info/pediatric/refvalues/reference.php .

¶ Values from patients of the present study.

n.s. : not significant; n.a. : not available.

Data of Table 3 confirm that red cells number, Hemoglobin, HCT, Neutrophils %, Lymphocytes %, MCP-1 and MIP-1ß serum values change linearly with age in IH samples but not in healthy age-matched samples, suggesting that their level may associate with the clinical IH phase.

### ROC analysis

**Figure 1:**
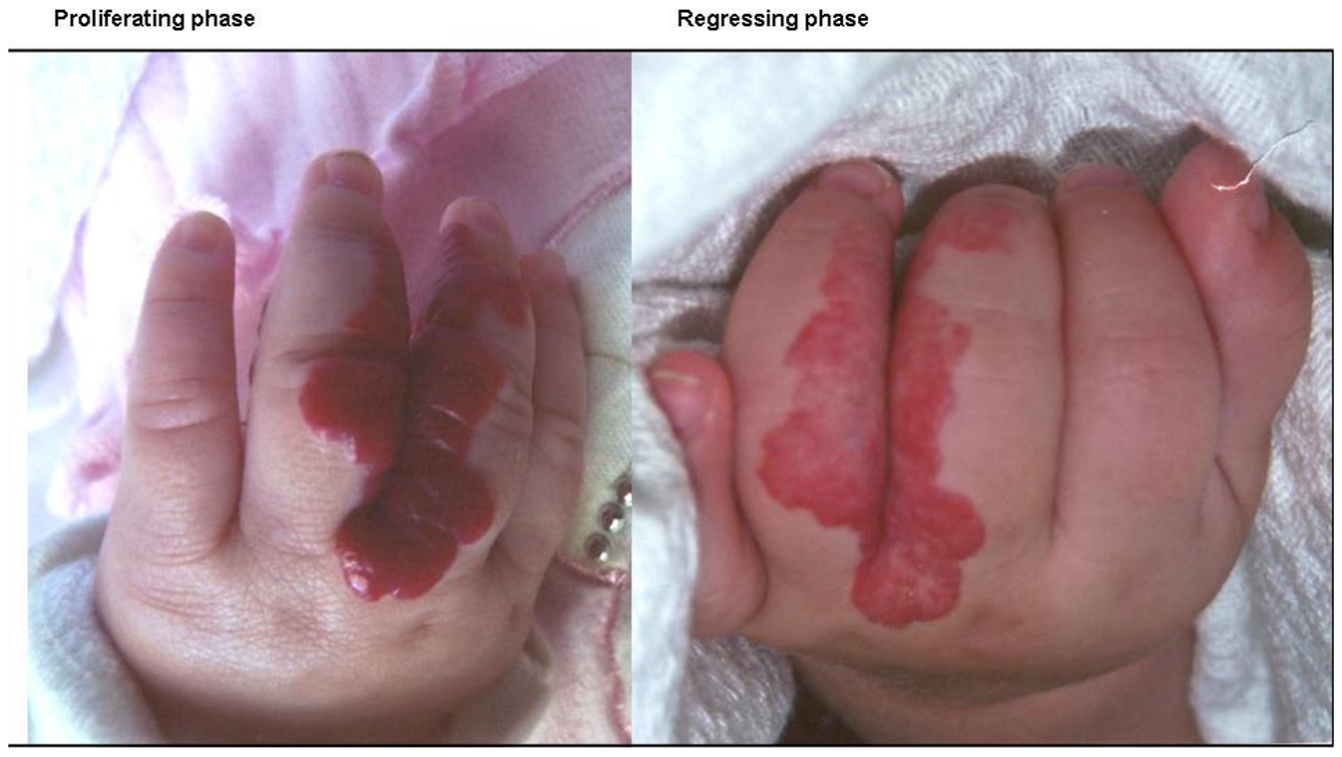
Representative image of IH at the proliferating phase (at month 6) and at regressing phase (at month 24).

According to data reported above, some serum values do not change in healthy blood-samples while they significantly change in IH blood-samples, likely representing signs of disease regression. In order to further test this hypothesis, ROC analysis was carried out, to assess whether such hematological values effectively discriminate individuals with proliferating IH (<12 months) from individuals with regressing IH (>12 moths). Fig. 1 shows a representative example of IH in the proliferating phase and in the regressing phase.

**Figure 2:**
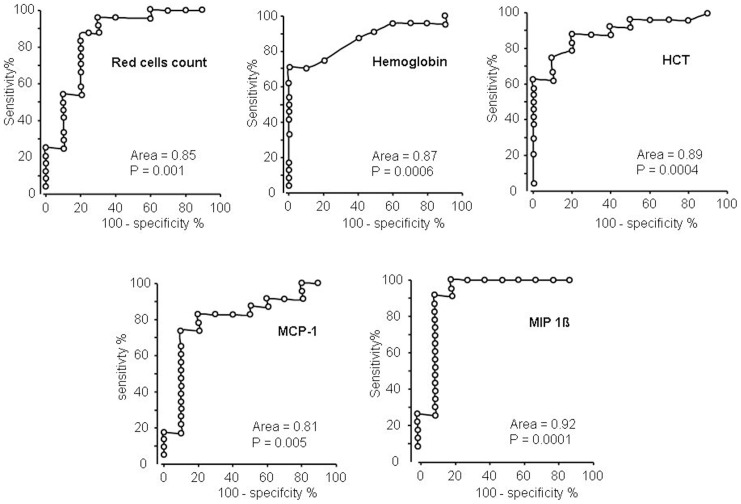
ROC analysis indicating that red blood cells, Hemoglobin, HCT, MCP-1 and MIP-1ß values significantly discriminate proliferating-IH samples (age≤12 months) from regressing -IH samples (>12 months age).

The computed area under the ROC curve indicates the ability to discriminate one group from the other taking into account both sensitivity and specificity. A value of 1 indicates the ability to discriminate 100% of individuals of one group from individuals of the other. Such analysis was carried out on all 46 measured parameters: red cells count, Hemoglobin, HCT, MCP-1 and MIP-1ß were the only parameters able to significantly discriminate proliferating IH from regressing IH showing a ROC area >0.80 and a strongly significant *p* value (Fig. 2).

## Discussion

A general aim of this study was to investigate whether standard hematological parameters or the cytokine serum profile may help identification of signs or mechanisms underlying the spontaneous regression of vascular tumors (namely IH). This may help clarifying, at least to some extent, the molecular machinery involved in tumor angiogenesis and cancer growth. IH is an interesting model of regression, since it regresses spontaneously as function of age, usually. In addition, babies show a lower dilution of serum factors given the smaller blood volume as compared to adults. Therefore, clarifying mechanisms underlying IH spontaneous regression may be of relevance for other human diseases showing deregulated angiogenic processes (e.g. solid tumors, diabetic retinopathy and myocardial infarction).

In the present pilot study routine diagnostic blood tests and non-standard measurements (namely cytokine/chemokines levels) were carried out in IH babies at proliferating and regressing phases. Clinical regression represents a key event allowing to rule out vascular malformations or other more malignant lesions, therefore identification of early molecular markers of regression may be useful to perform early differential diagnosis.

**Figure 3:**
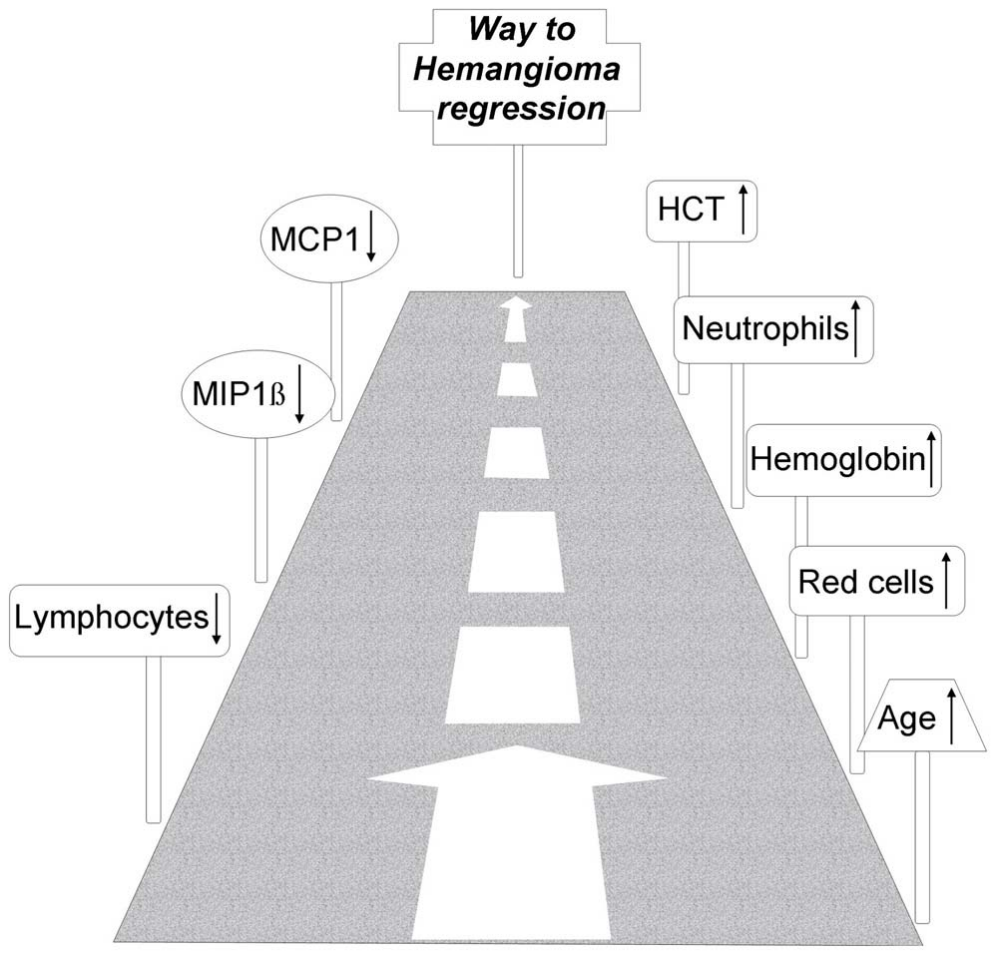
Cartoon summarizing the parameters characterizing IH regression.

Blood samples from babies with a clinical IH diagnosis were collected and spontaneous regression over time was observed in all selected cases. Hematological values were profiled according to routine diagnostic analyses and a number of interesting and significant changes was found, summarized in the cartoon reported in Fig. 3. The significant increase of red blood cells, Hemoglobin and HCT in involuting IH was rather interesting. In the involuting phase, reduction of the vascular lesions occurs; diseased vessel undergoes vasoconstriction leading to a slow progressive regression. Propanolol, a non-selective inhibitor of ß(1)- and ß(2)-adrenoceptor, is known to show a good therapeutic effect [8], [9], likely due, at least in part, to its ability to induce such vasoconstriction. In small babies vessel reduction occurring during the IH involuting phase may give a significant reduction of the vascular bed, relevant as compared to the body mass, with a consequent blood-concentration. This may explain to some extent the observed strong and significant increase of red blood cells, Hemoglobin and HCT as well as of Neutrophils %. However, blood concentration may not explain other unchanged or decreased hematological values. Interpretation of such data should take into account the half life of different molecules and cells. Red blood cells have 13 to 21 weeks life span [19], [20], while platelets last about 5 days [21]. Therefore blood cells content at a given time depends on the complex interaction of blood volume, cells half life and expression of chemokines with either pro- or anti- proliferating/chemoattractant/activating features. Therefore response latency to soluble factors may be very different for different cells. PDGF-BB is one of the growth factors known to be directly involved in the hemangioma onset and to inhibit hemangioma involution [22] along with FGF-2, a known potent regulator of endothelial cells and mast cells, both strongly involved in hemangioma onset [23]. Our group has previously shown the complex PDGF-BB/FGF-2 network being likely involved in the angiogenesis balance [24]–[27]. Data reported in Table 3 indicate a relevant (although not significant) variation of FGF-2 expression (more than 2 fold increase) counteracted by strong but less relevant change of PDGF-BB and VEGF, indicating a strong change of the PDGF-BB/FGF-2/VEGF balance and of their angiogenic potential.

Chemokines in general, and MCP-1 in particular, are known to control vessel distribution and branching points; their direct targets such as monocytes and macrophages have been called “architects” of vessel development [28]. The strong and significant decrease of serum MCP-1 and MIP-1ß was observed in IH involuting samples, either in ANOVA age-grouped analysis (Table 2) and in ungrouped samples (Table 3) and in ROC analysis (Fig. 2). MCP-1 action may underlie the observed effects on circulating cells. It is a chemoattractant molecule and represents the main stimulus for macrophage chemotaxis. MCP-1 activates monocytes, T cells and basophils [29] and belongs to the MCP (Monocyte Chemoattractant Proteins) family, a CC chemokine family, signalling via the CCR2 receptor. MCP-1 mRNA has been previously shown to be elevated in hemangioma tissues [30] and related to hemangioma onset [31]. Gordillo et al [31] carried out *in vitro* studies onto mouse EOMA endothelial cells, relating the endothelial response to their redox state. More recently Greenberger and colleagues [32] showed that MCP-1 is differentially expressed in proliferating *vs* regressed tissue IH specimens, by RT-qPCR, suggesting a role of NF-κB and VEGF-A in the IH pathogenesis. MCP-1 mRNA was found to be clearly reduced in 3 cases of involuting IH as compared to 6 proliferating cases. As compared to such previous data, our study is carried out on a larger sample size, reports a serum reduction of MCP-1, rather than a mRNA tissue reduction, and evidences the reduction at an earlier stage (mean age of the regressing IH in the present study is 34 months, *vs* 40 months mean age in the Greenberger study), supporting the conclusion that MCP-1 may represent a relevant early non invasive marker of regression. Given the observed spontaneous early modification and the known functional features of MCP-1, we hypothesize that MCP-1 besides a marker of regression, may also represent a potential therapeutic target to induce IH regression and control of vascular growth in other more serious angiogenic disorders. Anthocyanins antioxidant molecules such as OptiBerry have been shown [33] to exert clear anti-hemangioma effects and to reduce MCP-1 levels, further supporting the potential role of MCP-1 and the hypothesis that the redox state may represent a key mechanism controlling IH regression. Early molecular indicators of angiogenesis regression, such as MCP-1 as shown in Fig. 2, may represent putative therapeutic targets in other serious conditions showing deregulated angiogenesis, such as diabetes. In fact, MCP-1 role in cardiovascular diseases and diabetic retinopathy has been recently reviewed [34] and blood levels such as glycaemia have been directly related to pro-angiogenic features of circulating factors [35].

The other key factor found to be strongly changed in the present study is the CC chemokine Macrophage inflammatory protein-1β (MIP-1β), the specific ligand of CCR5 receptor. It is chemoattractant for monocytes and natural killer cells and induces monocytes adhesion to endothelial cells in atherosclerosis [36], [37]. MIP-1ß is involved in the transendothelial migration of *in vitro* cultured dendritic cells [38], [39] and is known to maintain the inflammatory/angiogenesis balance. Inflammation and angiogenesis are under the control of several common molecular mechanisms; inflammation can stimulate angiogenesis and, on the other hand, may facilitate inflammation. Either mechanisms have been shown to be involved in carcinogenesis. MIP-1ß is up-regulated in blood vessel tumors and its contribution to endothelial tumor initiation and/or progression has been indicated [40]–[42]. For instance MIP-1ß expression is known to characterize hemangiosarcomas (a tumor with an endothelial origin) while it is absent in urothelial carcinomas, i.e., an epithelial-origin tumor. In fact, in hemangiosarcoma MIP-1ß expression correlates with infiltrating leukocytes, tumor proliferation index and tumor angiogenesis [43]. All together these studies may confirm the hypothesis coming from the present study, suggesting MIP-1ß as a marker of IH regression and as key factor to control the angiogenesis balance.

Strong and significant differences were identified within the samples divided in three age-groups (≤12 month age, 13 to 24 months and >24 age) (see Table 1 and Table 2). Such differences were mostly confirmed when a correlation with the time as a continuous variable was used (right hand side in Table 3). This indicates that such parameters vary linearly with the age in IH samples (all showing spontaneous regression with time), while the same parameters do not show any correlation with age in healthy samples taken form the literature (left hand side in Table 3).

Finally, ROC analysis mostly confirmed these parameters as effective values to discriminate early (proliferating) IH from late (regressing) IH, indicating a potential diagnostic/prognostic application of such measures.

In the present study we show for the first time i) the significant change of several blood parameters as early serum indicators of IH regression and ii) potentially useful signs to monitor vascular growth occurring in other diseases such as solid tumors and diabetic retinopathy.

## Supporting Information

Table S1 **Clinical characteristics of the samples under study.**
(DOC)
