# Supplementary material for: Identification of Serum Regression Signs in Infantile Hemangioma
Source: PLoS One. 2014 Mar 5;9(3):e88545. doi: 10.1371/journal.pone.0088545 (PMC3943717; doi:10.1371/journal.pone.0088545)
Supplement: Table S1 — Clinical characteristics of the samples under study. (DOC) [file pone.0088545.s001.doc]

|  |  | |  |
| --- | --- | --- | --- |
| **Table S1. Clinical characteristics of the samples under study** | | | |
|  | **Group size** | | **Age* (months)** |
|  |  | |  |
| All | 34 | | 20 ± 4 |
| Male | 11 | | 18 ± 7 |
| Female | 23 | | 20 ± 4 |
|  |  | |  |
| Age at collection |  | |  |
|  |  | |  |
| ≤12 months | 10 | | 6 ± 1 |
| 13 to 24 months | 12 | | 19 ± 1 |
| >24 months | 12 | | 46.5± 6 |
|  |  | |  |
| * Average ± Standard Error | |  |  |
